# Insights from lncRNAs Profiling of MIN6 Beta Cells Undergoing Inflammation

**DOI:** 10.1155/2016/9275106

**Published:** 2016-09-06

**Authors:** Chuntao Sun, Lihua Xue, Ziyang Zhu, Fan Zhang, Ruixue Yang, Xuewen Yuan, Zhanjun Jia, Qianqi Liu

**Affiliations:** ^1^Department of Endocrinology, Nanjing Children's Hospital Affiliated to Nanjing Medical University, Nanjing 210008, China; ^2^Department of Child Health Care, Huaian Maternity and Child Healthcare Hospital Affiliated to Yangzhou University Medical College, Huaian 223002, China; ^3^The Fourth School of Clinical Medicine Nanjing Medical University, Nanjing 210029, China; ^4^Nanjing Key Lab of Pediatrics, Nanjing Children's Hospital Affiliated to Nanjing Medical University, Nanjing 210008, China; ^5^Department of Internal Medicine, University of Utah, Salt Lake City, UT, USA

## Abstract

Type 1 diabetes mellitus (T1DM) is an organ-specific autoimmune disease characterized by chronic and progressive apoptotic destruction of pancreatic beta cells. During the initial phases of T1DM, cytokines and other inflammatory mediators released by immune cells progressively infiltrate islet cells, induce alterations in gene expression, provoke functional impairment, and ultimately lead to apoptosis. Long noncoding RNAs (lncRNAs) are a new important class of pervasive genes that have a variety of biological functions and play key roles in many diseases. However, whether they have a function in cytokine-induced beta cell apoptosis is still uncertain. In this study, lncRNA microarray technology was used to identify the differently expressed lncRNAs and mRNAs in MIN6 cells exposed to proinflammatory cytokines. Four hundred forty-four upregulated and 279 downregulated lncRNAs were detected with a set filter fold-change ≧2.0. To elucidate the potential functions of these lncRNAs, Gene Ontology (GO) and pathway analyses were used to evaluate the potential functions of differentially expressed lncRNAs. Additionally, a lncRNA-mRNA coexpression network was constructed to predict the interactions between the most strikingly regulated lncRNAs and mRNAs. This study may be utilized as a background or reference resource for future functional studies on lncRNAs related to the diagnosis and development of new therapies for T1DM.

## 1. Introduction

Extensive epidemiological investigations have indicated that the incidence of type 1 diabetes mellitus (T1DM) in children and adolescents is increasing steadily year by year and is presumably to be doubled by 2020 [1–5]. However, the etiology and pathogenesis of T1DM are complex and have not been fully elucidated. Additionally, long-term insulin injection and acute and chronic clinical complications such as ketoacidosis, nephropathy, neuropathy, and retinopathy could cause significant physical and mental trauma on the children with this disease. Therefore, exploring the pathogenesis of T1DM and developing novel intervention strategies have become top priorities in the field of endocrine research.

Pancreatic beta cells secrete insulin to regulate blood glucose levels within a relatively narrow range. Research indicates that T1DM pathogenesis is associated with pancreatic beta cell apoptosis [6]. T1DM is an organ-specific autoimmune disease characterized by chronic and progressive apoptotic destruction of pancreatic beta cells, resulting in severe insulin deficiency [7]. Autoimmunity against the beta cells is likely triggered by environmental risk factors that act in genetically susceptible individuals [7–9]. Once stimulated, various immune cells, including T or B cells, macrophages, and dendritic cells, infiltrate the islets to induce beta cell death via mechanisms such as reactive oxygen species, perforin/granzymes, Fas/FasL, nitrogen species, and proinflammatory cytokines [6], among which cytokines have been recognized to play key roles in the regulation of autoimmunity and beta cell loss [10]. Prolonged exposure to immune cell-secreted proinflammatory cytokines such as interleukin- (IL-) 1*β*, tumor necrosis factor- (TNF-) *α*, and interferon- (IFN-) *γ* is highly cytotoxic to pancreatic beta cells, which impairs insulin secretion and ultimately induces beta cell loss by apoptosis [10–13]. During this inflammatory process, proinflammatory cytokines affect the expression of many gene networks and modulate pro- and antiapoptotic pathways [14, 15].

To date, research has mainly focused on protein-coding genes, though recent studies have shown that protein-coding genes account for less than 2% of the actively transcribed genome, suggesting that the majority of transcripts are noncoding RNAs (ncRNAs) [16]. NcRNAs can be subdivided by length into small ncRNAs (shorter than 200 nucleotides) and long ncRNAs (lncRNAs), which are greater than 200 nucleotides in length, with little or no protein-coding capacity [17]. In recent years, research in the field of RNA biology has mainly focused on small ncRNAs [18], largely ignoring lncRNAs. For a long time, these lncRNAs have been considered transcriptional noise. However, more and more evidence suggests that lncRNAs play significant roles in genome regulation, such as in X chromosome inactivation, genomic imprinting, chromatin modification, transcription, splicing, translation, degradation, and transport processes that are involved in the regulation of the growth and development of individual cells in apoptosis, proliferation, differentiation, and other cellular activities [17, 19–22]. The dysregulation of these lncRNAs has been associated with many human diseases, including different types of cancers, neurodegenerative diseases, and other disorders [23]. With the development of lncRNA microarrays, bioinformatics, and high-throughput sequencing, thousands of lncRNAs have been identified, though only a few of their features have been annotated. Currently, some studies have examined the roles of lncRNAs in diabetes [24], and the emerging evidence indicates that lncRNAs may be involved in maintaining pancreatic beta cell function and insulin signal transduction, which may affect diabetes development [25].

In this study, we examined the expression profiles of lncRNAs and mRNAs in MIN6 cells after exposure to a combination of IL-1*β*, TNF-*α*, and IFN-*γ* using microarray technology. We found that the expression levels of many lncRNAs and mRNAs changed in response to proinflammatory cytokines. Some of the interested lncRNAs with remarkable regulation were further verified by qRT-PCR, and the Gene Ontology (GO) and pathway analyses were used to analyze the biological roles of these differentially expressed lncRNAs and mRNAs. Coding-noncoding gene coexpression networks were used to predict the potential interactions between these lncRNAs and mRNAs. This study might provide important insights into the pathogenesis of beta cell apoptosis.

## 2. Materials and Methods

### 2.1. Cell Culture and Treatment

Pancreatic MIN6 *β*-cells were grown in high-glucose Dulbecco's Modified Eagle Medium (DMEM) supplemented with 10% fetal bovine serum (FBS), 100 *μ*g/mL streptomycin, 100 *μ*g/mL penicillin, and 50 *μ*M *β*-mercaptoethanol at 37°C in an atmosphere of 5% CO_2_. MIN6 cells were seeded in 6-well plates to approximately 80% confluence and were then treated with or without 5 ng/mL IL-1*β*, 25 ng/mL TNF-*α*, and 25 ng/mL IFN-*γ* for 24 h.

### 2.2. RNA Extraction and Quality Control

Total RNA was extracted from the MIN6 cells using TRIzol reagent (Invitrogen, USA) and was then purified using an RNeasy Mini Kit (Qiagen, Hilden, Germany) according to the manufacturer's protocol. RNA concentration and purity were determined with a OneDrop OD-1000. RNA integrity was measured using standard denaturing agarose gel electrophoresis.

### 2.3. Microarray and Data Analysis

#### 2.3.1. Mouse lncRNA Microarray

An Affymetrix GeneChip Mouse Transcriptome Array 1.0, which is designed for measuring a broad range of expression changes across the whole mouse transcriptome, was used to profile the ncRNAs and protein-coding genes. The lncRNAs were obtained from authoritative databases, including Ensembl, RefSeq, UCSC Known Genes, Vertebrate Genome Annotation (Vega) database, MGC Mammalian Gene Collection, MGI, NONCODE, and others.

#### 2.3.2. RNA Labeling and Array Hybridization

The sample preparation and labeling, microarray hybridization, and washing were performed using a GeneChip WT Terminal Labeling and Controls Kit, an Ambion WT Expression Kit, and a GeneChip Hybridization, Wash, and Stain Kit, respectively, according to the manufacturers' standard protocols. Briefly, 1 *μ*g total RNA, which was primed with primers containing a T7 promoter sequence, was used to synthesize single-stranded cDNA. Then, the single-stranded cDNA was transformed into double-stranded cDNA, which served as a template to synthesize and amplify the antisense RNA (complimentary RNA) from an in vitro transcription reaction. cRNA was reverse transcribed to sense-strand cDNA using 2nd-cycle primers. RNase H was used to hydrolyze the cRNA template, leaving single-stranded cDNA. After hydrolysis, the 2nd-cycle single-stranded cDNA, which was purified to remove salts, enzymes, and unincorporated dNTPs, was fragmented and labeled. The labeled cDNAs were hybridized onto the Affymetrix GeneChip Mouse Transcriptome Array 1.0 using a GeneChip Hybridization Oven 645. After washing the hybridized arrays, the arrays were scanned with a GeneChip Scanner 3000 7G.

#### 2.3.3. Data Analysis

Affymetrix® Expression Console*™* Software (version 1.3) was used to analyze the microarray data. The significant differentially expressed lncRNAs and mRNAs between the two groups were selected if the fold changes of the threshold values were ≧2.0 or ≦−2.0 (*P*≦0.05). The three-step Robust Multichip Analysis (RMA) algorithm, which includes (1) background adjustment, (2) quantile normalization, and (3) summarization, was used to analyze the raw data. Hierarchical clustering was performed using Cluster software 3.0. Microarray analysis was performed by Genminix Informatics Ltd., Co., China.

### 2.4. Quantitative Real-Time Reverse Transcription PCR (qRT-PCR)

Reaction cDNA was synthesized using the PrimeScript*™* RT Master Mix (Perfect Real Time, TAKARA, Japan) according to the manufacturer's recommendations. Reverse transcription of lncRNAs was performed using the SYBR green method in an Applied Biosystems 7500 system (Life Technologies, US). The gene expression levels were quantified based on the cycle threshold (CT) values and were normalized to the internal control gene *β*-actin. The relative expression levels of selected lncRNAs were calculated based on the 2^−ΔΔCT^ method. The primer sequences are listed in Table 1.

### 2.5. GO and Pathway Analyses

GO, which describes gene and gene product attributes, contains three key domains named biological process, cellular component, and molecular function. GO analysis was used to relate the differentially expressed mRNAs to GO categories. The Kyoto Encyclopedia of Genes and Genomes (KEGG) database, a bioinformatics resource for deciphering the genome, was used to identify the significant pathways of the targeted genes.

### 2.6. LncRNA-mRNA Coexpression Network

An lncRNA-mRNA coexpression network, which can identify the interactions between the differentially expressed lncRNAs and mRNAs, was constructed according to the normalized signal intensities of specifically expressed genes and lncRNAs. The Pearson correlation was calculated for each lncRNA-gene, gene-gene, or lncRNA-lncRNA pair, and significant correlation pairs were selected to construct the network.

### 2.7. Statistical Analysis

IBM SPSS 20.0 software was used to analyze all of the statistical data. The random variance model *t-*test was employed to identify the differentially expressed genes and lncRNAs between the control and cytokine-stimulated groups. Fisher's exact and *χ* ^2^ tests were applied for the GO and pathway analyses. *P* values < 0.05 were considered statistically significant.

## 3. Results

### 3.1. Microarray Data Profile

**Figure 1:**
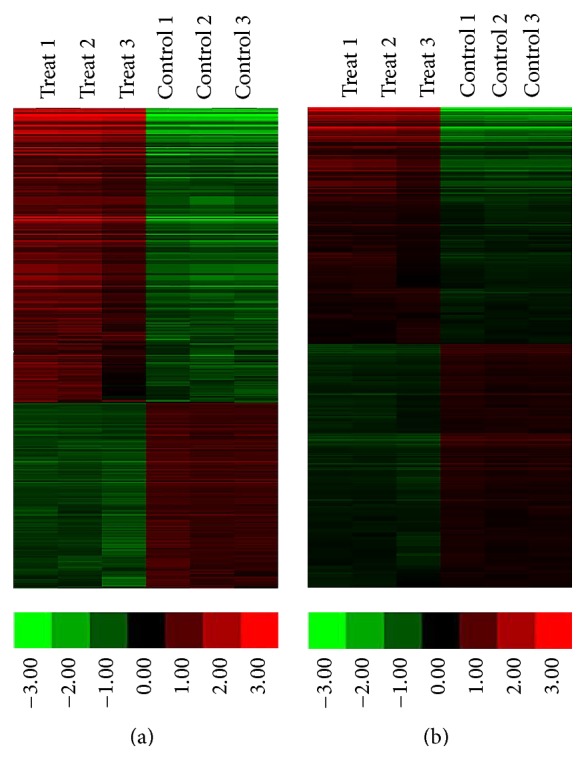
Profile of microarray data. (a) Hierarchical clustering shows a distinguished lncRNAs expression profiling among groups. (b) Hierarchical clustering shows a distinguished mRNAs expression profiling among groups. The red and the green shades indicate the expression above and below the relative expression, respectively, across all samples.

An Affymetrix GeneChip Mouse Transcriptome Array 1.0 was designed to profile all mouse lncRNAs and protein-coding transcripts. According to the microarray expression profiling data, 723 differently regulated lncRNAs were identified in the cytokine-stimulated group compared with the control group with a set filter fold-change ≧2.0, and 2180 differently regulated mRNAs were identified with a set filter fold-change ≧1.5. Additionally, 444 upregulated and 279 downregulated lncRNAs were detected and presented in table (in Supplementary Material available online at http://dx.doi.org/10.1155/2016/9275106). The result of the hierarchical clustering shows the lncRNA and mRNA expression patterns (Figures 1(a) and 1(b)).

### 3.2. LncRNA Classification and Subgroup Analysis

**Figure 2:**
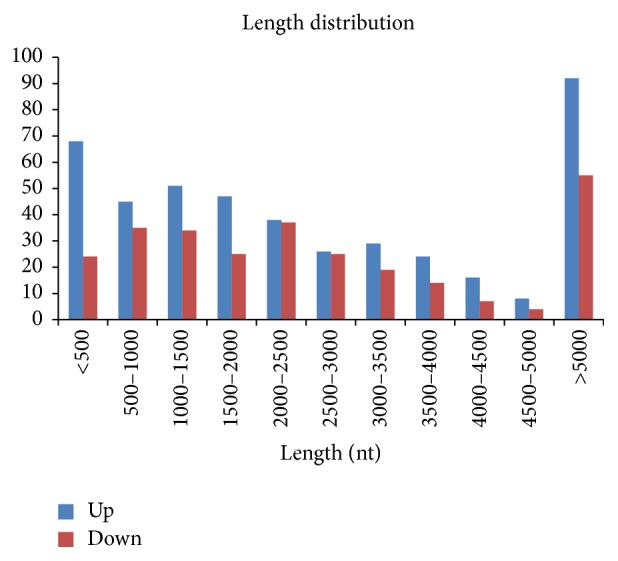
The length distribution of dysregulated lncRNAs. The lncRNAs were mainly less than 500 and longer than 5000 bp.

**Figure 3:**
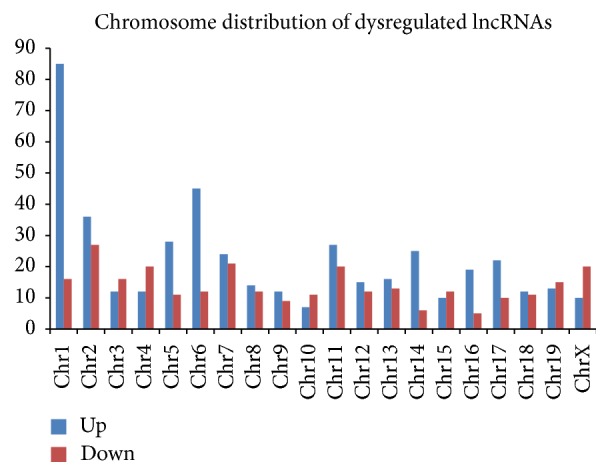
Chromosome distribution of up- and downregulated lncRNAs location in different chromosomes.

To further explore the potential functions of dysregulated lncRNA in the cytokine-stimulated group, we investigated their lengths and chromosomal distributions. The length distribution showed that the deregulated lncRNAs were mostly less than 500 bp or longer than 5000 bp (Figure 2). The chromosomal distribution showed that the upregulated lncRNAs were mostly located on chromosome 1 and that the downregulated lncRNAs were mostly located on chromosome 2 (Figure 3).

### 3.3. GO and Pathway Analyses

**Figure 4:**
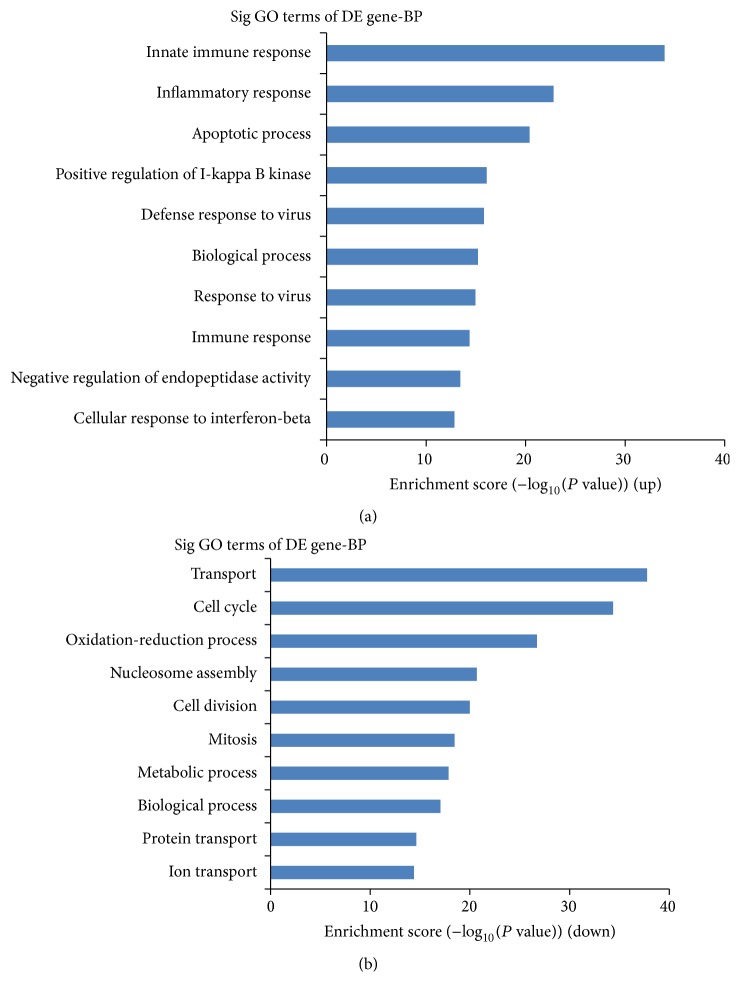
GO analyses. (a) The top 10 GO terms that associated with coding gene function of upregulated lncRNAs are listed. (b) The top 10 GO terms that associated with coding gene function of downregulated lncRNAs are listed.

Based on our GO analysis, the following top 10 enriched GO terms were associated with the upregulated lncRNAs: (1) innate immune response, (2) inflammatory response, (3) apoptotic process, (4) positive regulation of I-kappa B kinase/nuclear factor- (NF-) kappa B cascade, (5) defense response to virus, (6) biological process, (7) response to virus, (8) immune response, (9) positive regulation of transcription from RNA polymerase II promoter, and (10) negative regulation of endopeptidase activity (Figure 4(a)). The following top 10 enriched GO terms were associated with the downregulated lncRNAs: (1) transport, (2) cell cycle, (3) oxidation-reduction process, (4) nucleosome assembly, (5) cell division, (6) mitosis, (7) metabolic process, (8) biological process, (9) protein transport, and (10) ion transport (Figure 4(b)).

**Figure 5:**
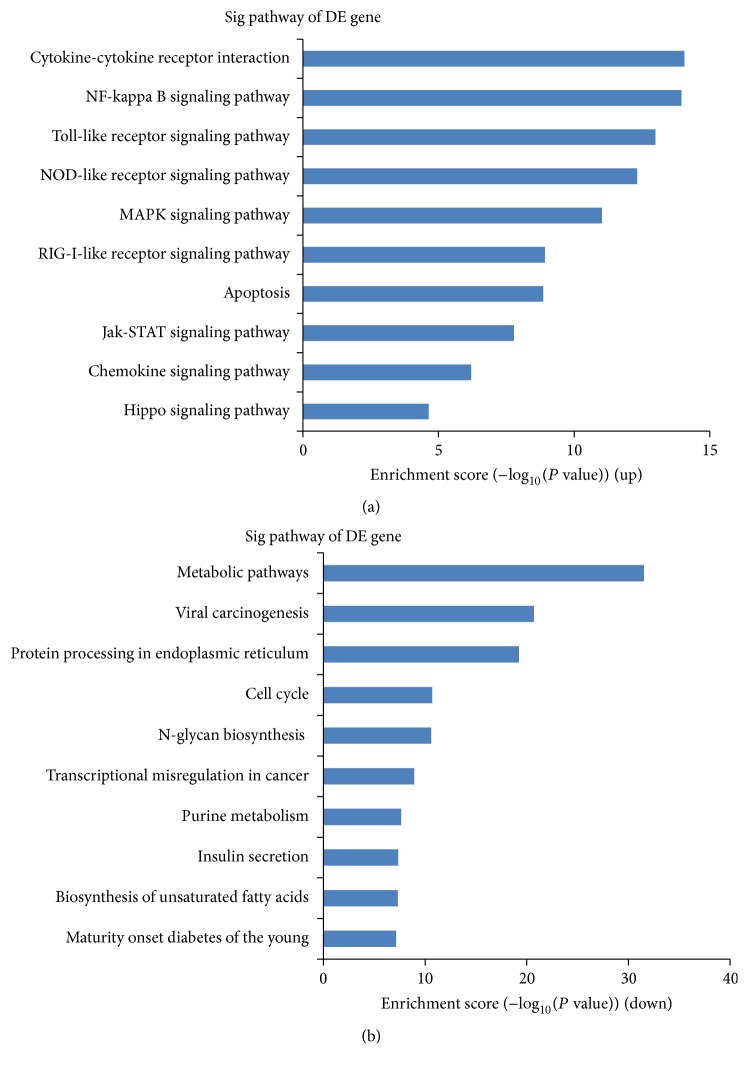
Pathway analyses. (a) The top 10 pathways that associated with coding gene of upregulated lncRNAs are listed. (b) The top 10 pathways that associated with coding gene of downregulated lncRNAs are listed.

Based on our pathway analysis, the following top 10 enriched pathways were associated with the upregulated lncRNAs: (1) cytokine-cytokine receptor interaction, (2) NF-kappa B signaling pathway, (3) Toll-like receptor signaling pathway, (4) NOD-like receptor signaling pathway, (5) MAPK signaling pathway, (6) RIG-I-like receptor signaling pathway, (7) apoptosis, (8) Jak-STAT signaling pathway, (9) chemokine signaling pathway, and (10) Hippo signaling pathway (Figure 5(a)). Additionally, the following top 10 enriched pathways were associated with the downregulated lncRNAs: (1) metabolic pathways, (2) viral carcinogenesis, (3) protein processing in endoplasmic reticulum, (4) cell cycle, (5) N-glycan biosynthesis, (6) transcriptional misregulation in cancer, (7) purine metabolism, (8) insulin secretion, (9) biosynthesis of unsaturated fatty acids, and (10) maturity onset diabetes of the young (Figure 5(b)).

### 3.4. Construction of the lncRNA-mRNA Coexpression Network

**Figure 6:**
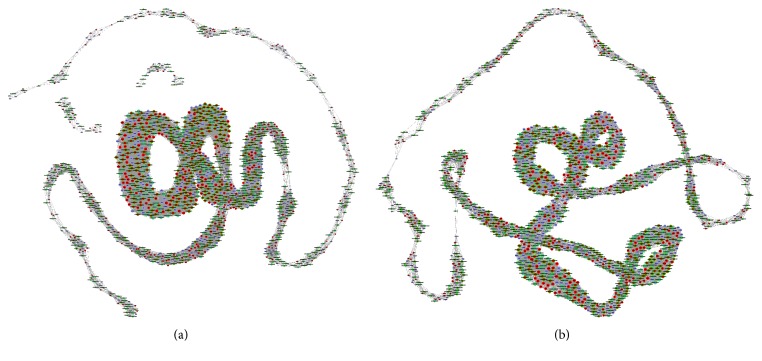
The lncRNA-mRNA coexpression network. (a) The lncRNA-mRNA network containing the 625 filtered mRNAs and 723 filtered aberrant expressed lncRNAs in cytokine stimulation group. (b) The lncRNA-mRNA network containing the 625 filtered mRNAs and 723 filtered aberrant expressed lncRNAs in control group. Upregulated RNAs are shown in red, and downregulated RNAs are presented in purple. Nodes without a ring represent mRNAs, nodes with a ring represent lncRNAs, and node size represents the degree of centrality.

Based on the correlations between the differentially expressed lncRNAs and mRNAs with significant correlation Pearson coefficients, we developed a profile between the lncRNAs and coding mRNAs in the cytokine-stimulated and control groups (Figures 6(a) and 6(b)). The coexpression network showed that one lncRNA was associated with one to dozens of mRNAs and lncRNAs. In a network analysis, degree centrality is used to measure a gene or lncRNA centrality within a network, determining its relative importance. From the two networks, we identified the core regulatory lncRNAs and mRNAs, which had the largest degrees (Tables 2 and 3).

### 3.5. Real-Time Quantitative PCR Validation

**Figure 7:**
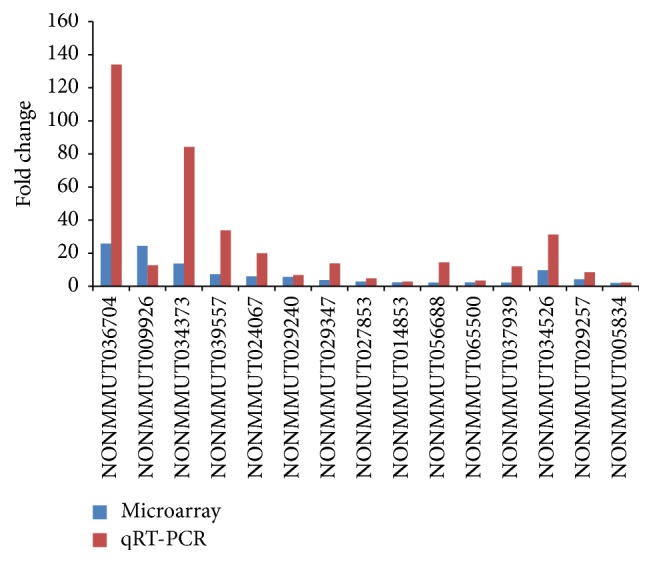
Comparison between microarray data and qRT-PCR result for lncRNAs. The validation results indicated that the microarray data correlated well with the qPCR results.

We randomly selected 15 interested lncRNAs to validate the microarray analysis data using qRT-PCR. The results of the qRT-PCR were largely consistent with the data from the microarray (Figure 7).

## 4. Discussion

During the initial phases of T1DM, cytokines and other inflammatory mediators released by immune cells progressively infiltrate islets, contributing to the functional suppression and death of beta cells [12]. In nonobese diabetic (NOD) mice, beta cell apoptosis is preceded by substantial lymphocytic infiltration [26]. These observations led to the concept that proinflammatory mediators produced by infiltrating cells play key roles in inducing beta cell death. Accordingly, prolonged exposure to proinflammatory cytokines is highly cytotoxic to pancreatic beta cell function [10–13] and affects the expression of many gene networks [14, 15]. Previous studies have mainly focused on the various miRNAs that regulate beta cell apoptosis. Little information exists on the lncRNAs that regulate beta cell apoptosis and contribute to T1DM development [27–29]. Compared with these studies, we use a new and different gene chip, expand the lncRNA expression profile, and update the database. Here, we report the lncRNA expression profiles of MIN6 cells that have been exposed to the proinflammatory cytokines IL-1*β*, TNF-*α*, and IFN-*γ*.

Comprehensive analysis shows that lncRNAs are generally expressed at lower levels compared with protein-coding genes and are most likely to display tissue-specific expression patterns [30, 31]. The Affymetrix GeneChip Mouse Transcriptome Array 1.0, which contains both lncRNA and mRNA probes, was employed to determine the expression profiles of lncRNAs and mRNAs in MIN6 cells. In this study, we identified 723 differently regulated lncRNAs and 2180 differently regulated mRNAs in the cytokine-stimulated group compared with the control cells, among which 444 upregulated and 279 downregulated lncRNAs were detected with a set filter fold-change ≧2.0. qRT-PCR was used to validate some of the microarray analysis data, and the results were consistent with the microarray data. These results suggest that the lncRNA expression signatures were unique in MIN6 cells.

The mostly upregulated lncRNA, NONMMUT036704, was found to exhibit sense overlap with the lipocalin 2 (Lnc2), which is also known as neutrophil gelatinase-associated lipocalin (NGAL). NGAL play an important role in a variety of diseases. Jung et al. found that IL-10 could mediate NGAL expression in breast cancer cells, which might contribute to tumor progression [32]. In addition, NGAL could create the local and systemic proinflammatory environment for atherosclerosis by induction of proinflammatory mediators [33]. And, NGAL was overexpression in subcutaneous adipose tissue in overweight women with gestational diabetes mellitus (GDM), which suggests that it may play a role in the development of insulin resistance in GDM [34]. Currently, there are no studies about NGAL in the apoptosis of *β* cells. But in view of its role in the parenchyma cells, we surmise that lncRNA NONMMUT036704 may play an important role in the development of T1DM through the regulation of NGAL. NONMMUT034373 is another highly upregulated lncRNA. This transcript was found to sense overlap CD274 antigen (Cd274), which is also known as programmed death-1 ligand-1 (PD-L1). PD-L1 is the ligand of PD-1. A recent study shows that forced expression of PD-1 in transgenic mice significantly decreased the incidence of autoimmune diabetes [35]. Another study also shows that PD-1 or PD-L1 blockade rapidly precipitated diabetes in prediabetic female nonobese diabetic (NOD) mice regardless of age [36]. PD-L1 deficiency could increase susceptibility to diabetes via a direct effect on pathogenic T cells, while regulatory T cells and B cells prevented autoimmune diabetes via a mechanism independent of PD-1/PD-L1 pathway [37]. Therefore, we surmise that lncRNANONMMUT034373 may contribute to the development of T1DM via regulating PD-L1.

Genetic predisposition is a major etiologic factor in T1DM development. Studies have reported that there are susceptible genes to T1DM in some chromosomes, such as PTPN22 on chromosome 1, CTLA4 on chromosome 2, IFIH1 on chromosome 2, and IL2RA on chromosome 10 [38–41]. In this study, compared with the other chromosomes, chromosome 1 had a higher percentage of upregulated lncRNAs, and chromosome 2 had a higher percentage of downregulated lncRNAs. This finding suggests that we may have a higher chance to find the susceptible lncRNAs that contribute to beta cell apoptosis on chromosomes 1 and 2.

To evaluate the potential biological functions of the differentially expressed lncRNAs, GO enrichment and pathway analyses were used to analyze the differentially expressed protein-coding genes associated with these lncRNAs. In our existing data, we found that the main biological processes that were enriched for the dysregulated lncRNAs were “innate immune response,” “inflammatory response,” “apoptotic process,” and “positive regulation of I-kappa B kinase/NF-kappa B cascade.” These findings are in agreement with one current concept that immune-mediated inflammation may serve as a pathogenic mechanism of type-1 diabetes. The released inflammatory cytokines bind to their receptors on beta cells and could activate the transcription factors p38, c-Jun N-terminal kinase (JNK), signal transducer and activator of transcription-1 (STAT-1), and NF-kappa B and the mitogen-activated protein- (MAP-) kinase signaling pathways, provoking endoplasmic reticulum stress and leading to functional impairment and ultimately apoptosis [6].

Pathway analysis is a functional analysis that maps genes onto KEGG pathways. Here the pathway analysis showed that the identified lncRNAs are mainly involved in the NF-kappa B signaling pathway, Toll-like receptor signaling pathway, MAPK signaling pathway, and Jak-STAT signaling pathway, indirectly indicating that these differentially expressed lncRNAs play important roles in cytokine-induced islet cell injury. Pathway analysis also showed that the identified lncRNAs are involved in the Hippo signaling pathway, RIG-I-like receptor signaling pathway. The Hippo signaling pathway plays critical roles in organ size control and tissue regeneration by inhibiting cell proliferation and promoting apoptosis. After partial hepatectomy (PH) in rats, the Hippo signaling pathway is inhibited early during liver regeneration [42]. In addition, inactivation of the Hippo pathway or activation of its downstream effector, the YAP transcription coactivator, improves cardiac regeneration [43]. Therefore, we assumed that activation of the Hippo signaling pathway may induce *β* cells apoptosis and inactivation of the Hippo signaling pathway may induce *β* cells proliferation. This could be a new therapeutic target for TIDM. RIG-I-like receptors are key cytoplasmic pathogen recognition receptors that are involved in the recognition of viruses, including functioning as major sensors of RNA viruses, and promoting recognition of some DNA viruses [44]. Although there are no evidence showing that RIG-I-like receptors are associated with type 1 diabetes, environmental factors such as virus infection have been thought to trigger T1DM. Therefore, we speculate that activation of RIG-I-like receptors may cause innate immune-related inflammatory response to promote *β* cells apoptosis, resulting in T1DM.

## 5. Conclusion

Our current study reveals many differentially expressed lncRNAs and their related mRNAs in cytokine-induced MIN6 cell apoptosis using a microarray assay. These differentially expressed lncRNAs may play key or partial roles in cytokine-mediated beta cell dysfunction. Understanding the functions of these lncRNAs could help us to identify new diagnostic and therapy targets for T1DM. Thus, further studies to identify the contributions of some interested targets in the pathogenesis of T1DM based on current study would be important and valuable.

## Supplementary Material

Differentially expressed lncRNAs.

## Figures and Tables

**Table 1:** Primers used for RT-qPCR analysis.

Primer name	Primer sequence (5′-3′) forward	Primer sequence (5′-3′) reverse
NONMMUT036704	CTGAATGGGTGGTGAGTGTG	GCTCTCTGGCAACAGGAAAG
NONMMUT009926	CACACCAGAAGAGGTCGTCA	GGCTCAATGGGTAAGAGCAC
NONMMUT034373	GGCTCTCAGCTCTTGTCTGG	AGCATGGCCTTTGACCTCTA
NONMMUT039557	GCTCACCACTCCTCTCTTCC	AAGAATGCAGGCAAACCCTA
NONMMUT024067	TCCAGAGATGATGGGTCCTT	ACAGTCCAGAGAGGCAGACC
NONMMUT029240	AAGAAACCTGCCACACTGCT	CGAAGACTCACCATCCACCT
NONMMUT029347	GTTTCCACCCATGTTGTGC	GCCTTTCTCCTACACCACCA
NONMMUT027853	CAATCAGGAGGGATCTTCCA	ACACGGAGCAGAAAGAGGAG
NONMMUT014853	GAGGCATCTGCTTGACTGTG	TTCGTGCTATGCTCACTGCT
NONMMUT056688	GCCCTGATACTGAGGAGTGG	TTTGTGCAGGAAGGGAAATC
NONMMUT065500	GGGAGAGGAAGGAGGTGTTC	AGGACCGCATACTCAGGAGA
NONMMUT037939	GATGAAGAAGCTGGACCTCAA	GGACGTTTGGAGGAAGGAAT
NONMMUT034526	GGATGAAGGGATTGCTGATT	GCTGTCTTCCTGGCATTTCT
NONMMUT029257	CCTCTATCCACCGACTCCAA	CACGCTGTGTTTCTCCTCCT
NONMMUT005834	CATAGATGGCACATGGGAAG	ATCTCACCTCTGGCACACAC
*β*-actin	AGAGGGAAATCGTGCGTGAC	CCATACCCAAGAAGGAAGGCT

**Table 2:** Part of lncRNAs and mRNAs owned the biggest degree in control group.

Gene symbol/lncRNA	Degree	Style	Type
Shmt2	55	Up	Coding
NONMMUT069313	54	Up	Noncoding
Vimp	54	Down	Coding
NONMMUT068653	54	Up	Noncoding
Asap3	53	Up	Coding
NONMMUT001721	53	Down	Noncoding
NONMMUT067923	53	Up	Noncoding
Flna	52	Up	Coding
XR_141418	52	Down	Noncoding
Ganab	52	Down	Coding

**Table 3:** Part of lncRNAs and mRNAs owned the biggest degree in cytokine stimulation group.

Gene symbol/lncRNA	Degree	Style	Type
NONMMUT001768	89	Up	Noncoding
Ap3s1	89	Down	Coding
NR_037617	89	Up	Noncoding
NONMMUT004294	88	Down	Noncoding
Usp1	88	Down	Coding
NONMMUT036704	88	Up	Noncoding
NONMMUT040558	88	Up	Noncoding
NONMMUT037904	88	Down	Noncoding
NONMMUT069313	87	Up	Noncoding
Cdc25c	87	Down	Coding
